# Krabbe Disease: Prospects of Finding a Cure Using AAV Gene Therapy

**DOI:** 10.3389/fmed.2021.760236

**Published:** 2021-11-11

**Authors:** Gibran Nasir, Rajiv Chopra, Fiona Elwood, Seemin S. Ahmed

**Affiliations:** ^1^Department of Neuroscience, Novartis Institutes for BioMedical Research (NIBR), Cambridge, MA, United States; ^2^AllianThera Biopharma, Boston, MA, United States

**Keywords:** Krabbe disease (globoid cell leukodystrophy), leukodystrophies, adeno-associated virus, gene therapy, galactocerebrosidase, psychosine

## Abstract

Krabbe Disease (KD) is an autosomal metabolic disorder that affects both the central and peripheral nervous systems. It is caused by a functional deficiency of the lysosomal enzyme, galactocerebrosidase (GALC), resulting in an accumulation of the toxic metabolite, psychosine. Psychosine accumulation affects many different cellular pathways, leading to severe demyelination. Although there is currently no effective therapy for Krabbe disease, recent gene therapy-based approaches in animal models have indicated a promising outlook for clinical treatment. This review highlights recent findings in the pathogenesis of Krabbe disease, and evaluates AAV-based gene therapy as a promising strategy for treating this devastating pediatric disease.

Krabbe Disease (KD) is a devastating pediatric lysosomal storage disorder that affects both the central and peripheral nervous systems. It was first described in detail in 1916 by a Danish neurologist, Knud Krabbe who also lends his name to the disease (1). This autosomal recessive disease is caused by the functional deficiency of the lysosomal enzyme, galactocerebrosidase (GALC). GALC degrades complex galactosides, carbohydrate molecules such as galactocerebrosides and galactosylsphingosine, to provide metabolites, such as sphingosine and ceramide, which are critical for the synthesis and maintenance of myelin, the insulating sheath that improves electrical transmission along neurons. The absence of GALC results in the accumulation of psychosine, which is toxic at high concentrations. Disease symptoms vary in severity, and are classified based on the age at which symptoms appear (2) (Figure 1).

**Figure 1:**
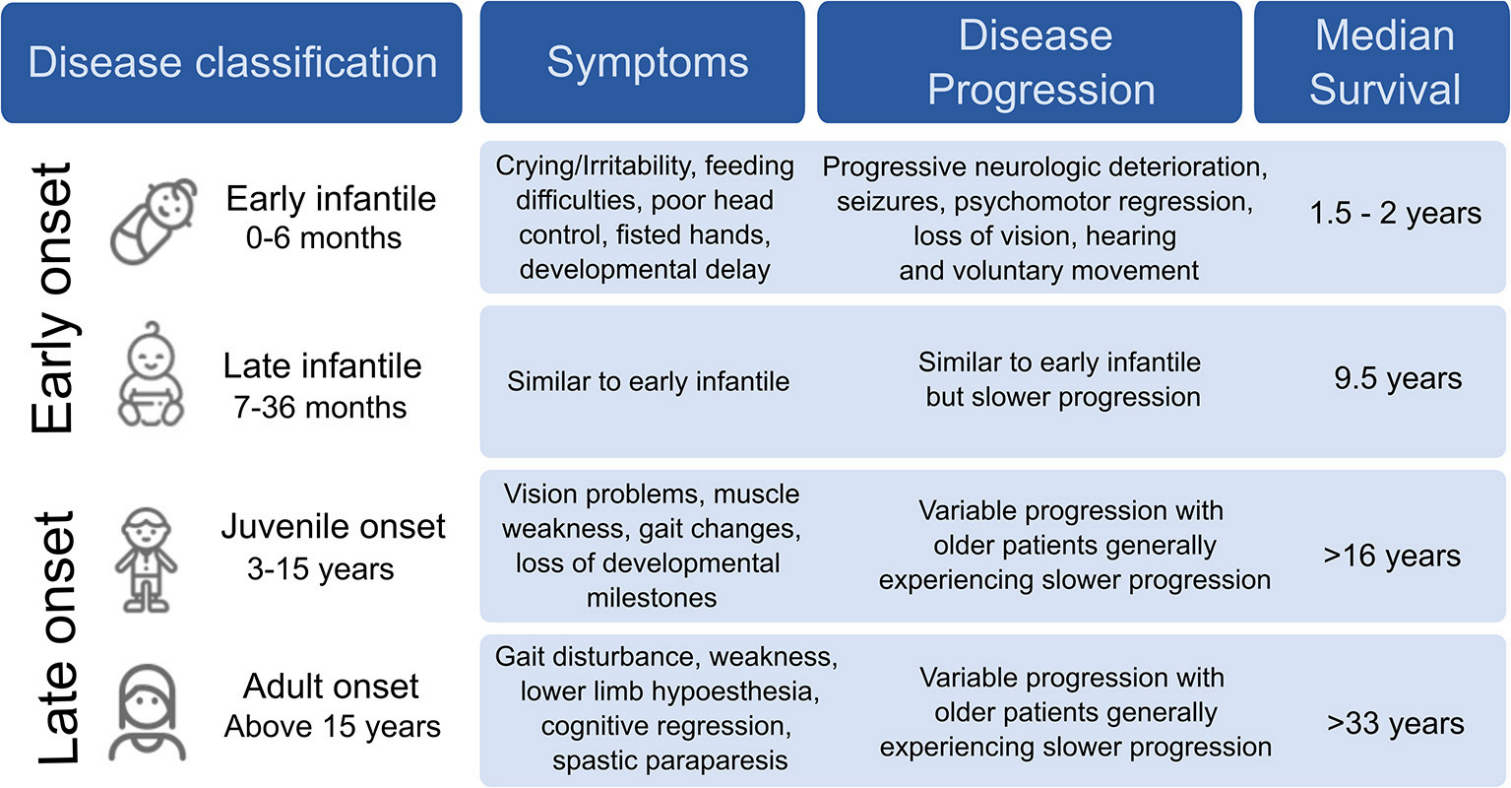
Clinical classification of Krabbe disease.

In infants, KD is generally diagnosed by 4 months of age, with death occurring within 2–3 years if left untreated, largely due to organ failure because of severe demyelination (3). Common symptoms include irritability, paralysis, blindness, hearing loss, and seizures. The severity of symptoms in early onset KD may result from a complete loss of GALC activity (2, 4). In late onset KD, the appearance of symptoms is more variable, with the oldest patient diagnosed at 72 years (5).

This review describes recent findings that provide insights into pathological mechanisms of KD and evaluates therapeutic avenues thus far explored in treating KD, with a focus on AAV-based gene therapies.

## Structure and Biochemistry of Galactosylcerebrosidase (GALC)

In humans, the GALC gene is located on chromosome 14 (14q31.3) and has 17 exons (6). The GALC protein comprises 669 amino acids and has six potential N-glycosylation sites that engage the mannose-6-phosphate (M6P) receptor for trafficking to lysosomes (7).

**Figure 2:**
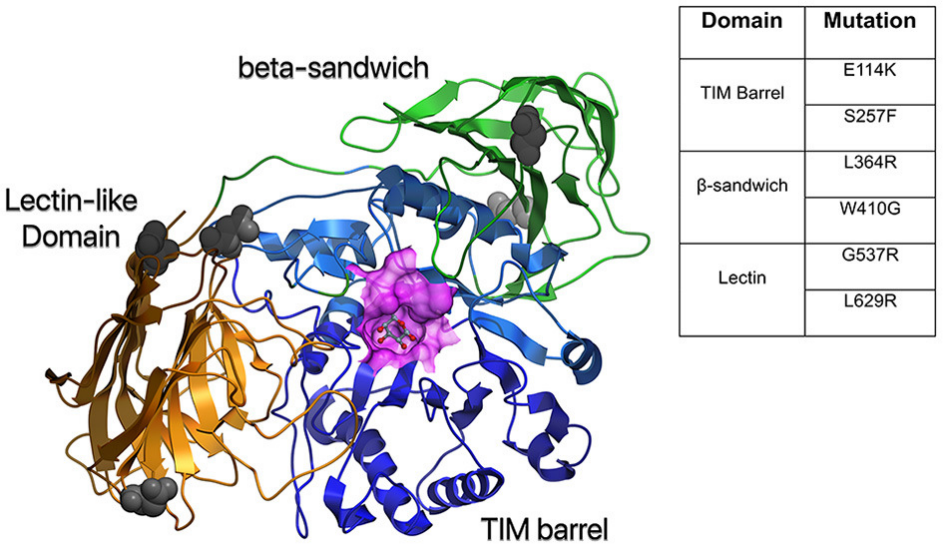
Structural model of human GALC. A homology model was created based on mouse GALC (RCSB:3ZR5) and colored according to domain: TIM barrel in blue, Lectin domain in gold, and β-sandwich domain in green. Also shown is a surface representation of the active site in purple and gray atoms to indicate putative glycosylation sites. The table shows common mutations in the different protein domains of GALC that result in severe disease.

The structure of GALC includes an overall fold that comprises three domains: a central triosephosphate isomerase (TIM) barrel, a β-sandwich domain, and a lectin domain that make up the GALC substrate-binding site (7). While the organization of human GALC has been described, the crystal structure is not yet available (8). The structure of mouse GALC (83% identity with human GALC), however, both alone and in complex with D-galactose, one of its products, has been solved (7) (Figure 2). This structure shows the unique domain arrangement of GALC, and identifies the nature of substrate binding. In addition, it highlights the site of catalysis and provides insight into the role of mutations occurring in human KD patients. Understanding how mutations impact the structure of GALC has important implications for the rational design of therapeutics such as pharmacological chaperones. Computational advancements in artificial intelligence and machine learning have improved our understanding of positional implications of mutations in the 3 dimensional structure of proteins with databases such as HOPE (9).

**Figure 3:**
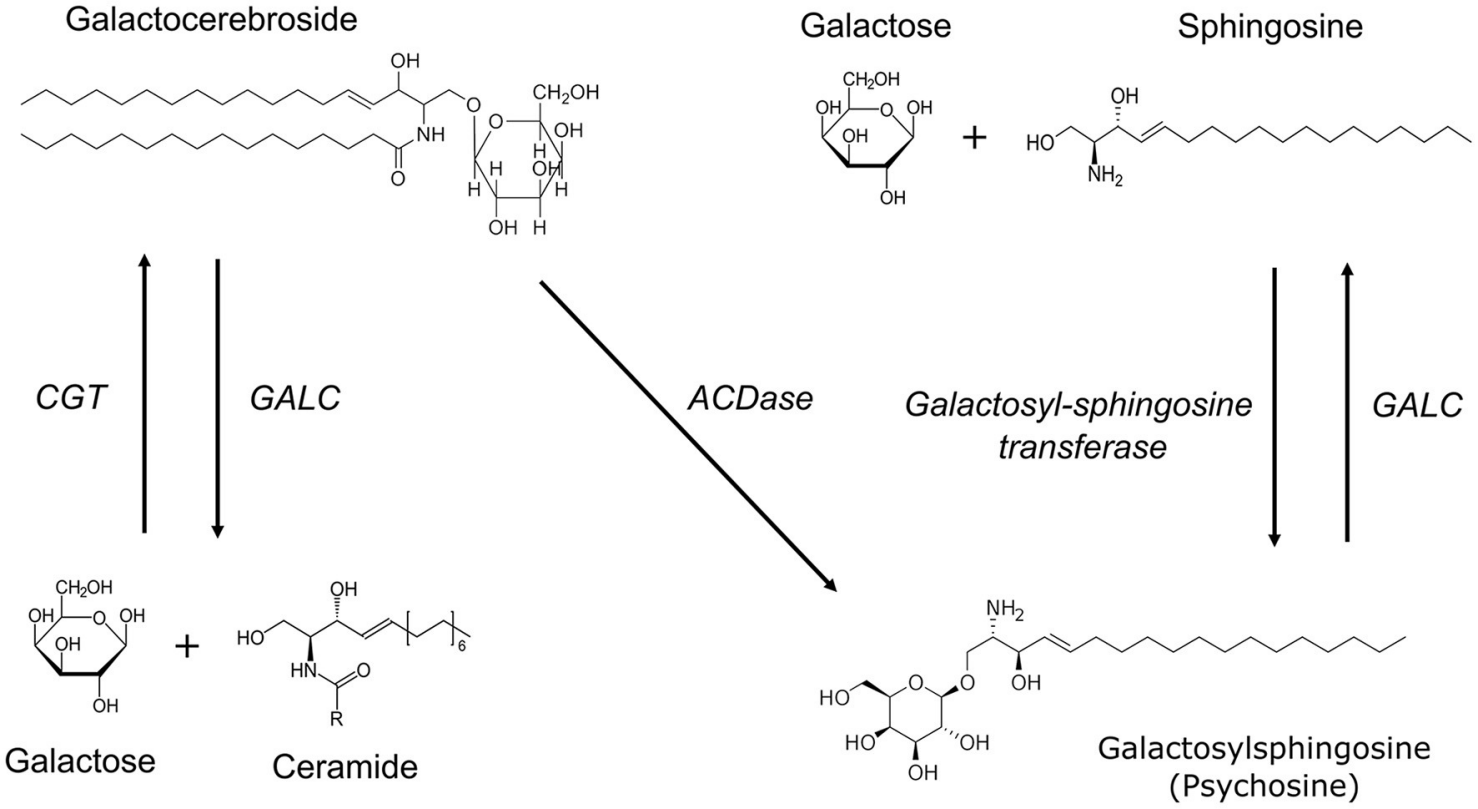
Biochemical pathways involved in myelin lipid synthesis and breakdown. UDP-galactose ceramide galactosyltransferase (CGT) catalyzes the synthesis of galactocerebrosides from galactose and ceramide. Galactocerebrosidase (GALC) is involved in the hydrolysis of both galactocerebroside and galactosylsphingosine (psychosine). Psychosine can be synthesized from UDP-galactose and sphingosine by the enzyme galactosyl-sphingosine transferase. In the absence of GALC, psychosine can also be generated directly from galactocerebrosides by acid ceramidase (ACDase).

GALC catalyzes the synthesis of galactose from glycosphingolipids like galactocerebroside and galactosylsphingosine (Figure 3). In the absence of GALC, a secondary β-galactosidase can catalyze the formation of galactose from galactosylceramide. In addition, psychosine can also be generated directly from galactocerebrosides by acid ceramidase (ACDase) (10), a lysosomal enzyme that is activated through various external stimuli including, but not limited to, Saposin D, TNFα, and IL-1β (11). Interestingly, GALC is the only enzyme that can degrade psychosine (10) thus its absence leads to a dysregulated accumulation of psychosine that is toxic at high concentrations.

Psychosine accumulation, however, is not unique to deficiencies in GALC. A small glycoprotein, Saposin A (SapA) enhances GALC activity through the formation of a heterodimeric complex with GALC where it helps to orient substrates for effective cleavage (12). Although rare, deficiency in SapA can also lead to clinical symptoms that phenocopy early onset KD (13). In SapA deficiency, GALC enzymatic activity is preserved but psychosine is elevated to levels generally found in symptomatic KD patients. These biochemical pathways are critical in myelin synthesis and homeostasis.

## Epidemiology, Genetics, and Biomarkers

Multiple epidemiological studies have been conducted on KD but the data has been difficult to reconcile due to the use of different study populations and varying methods of estimation (14). The accepted incidence of KD in Europe is 1:100,000 (15, 16) and in some communities with consanguineous marriages like the Druze and Muslim Arabs, the incidence of KD is estimated at 1:100 to 1:150 (17). Although previous studies suggest an estimated incidence of 1:250,000 in the USA (18), an epidemiological study done in New York state (NYS) using newborn screening data estimated the state-wide incidence to be 1:394,000 (19). A recent study in Spain unexpectedly found the prevalence of KD to be highest compared to several other lysosomal storage disorders, including Pelizaeus-Merzbacher disease and Sulfatide Lipidoses (20), indicating a need for accurate regional disease incidence and patient demographic studies. Currently, newborn screening with elevated psychosine as a marker is increasingly being used for detection of KD (15) though presently, only eight states in the USA include KD on their newborn screening panel.

To date, at least 147 mutations in GALC have been documented, with 80 of them considered to be “severe” due to their impact on GALC activity (4). These mutations can be missense mutations, non-sense mutations (premature stop codons), deletion mutations (particularly a 30 kb deletion that is found in 30–50% of infantile Krabbe disease cases), insertions, and frameshift mutations (4). While disease-associated mutations in GALC occur throughout the different domains, certain homozygous mutations result in more severe symptoms than others (Figure 2). Mutations such as E114K and S257F in the TIM barrel domain, L364R and W410G in the β-sandwich domain, and G537R and L629R in the lectin domain cause severe misfolding and likely result in early degradation of GALC (7). Some mutations, such as E215K, can cause severe disease by disrupting the interaction with auxiliary proteins, such as SapA, and impacting GALC function (7). Similarly, the D528N mutation (common among Muslim Arab populations in Israel), which introduces a new glycosylation site and impairs GALC localization to the lysosome, can lead to severe disease (21, 22). However, the correlation between GALC mutations and disease severity can be unpredictable since mutations are not always inherited in a homozygous manner.

Geographical differences in disease-causing mutations have also been reported. For instance, the missense mutations that are frequently found in KD patients in China (H253Y, S259L, P318L, F350V, T428A, L530P, and G586D) are different than those found in Europe and Japan (23). In Japan, the three most common missense mutations were found to be I66M + I289V, G270D, and T652P (24). Similarly, a study characterizing GALC mutations in European KD patients reported four missense mutations that included P318R, G323R, I384T, and Y490N (25). In the USA, based on the NYS referral population, the most common pathogenic mutations were T96A and Y303C, which frequently co-occurred with non-pathogenic enzyme reducing mutations R168C, D232N, and I546T (26).

In all cases and across species, pathogenic mutations in GALC are associated with a dramatic increase in levels of psychosine within the central nervous system (CNS) and peripheral nervous system (PNS). Although psychosine levels are a better biomarker for clinical diagnosis compared with GALC activity, there is some debate concerning its use as a presymptomatic biomarker. Hypoxanthine, a metabolite in the purine nucleotide salvage pathway is elevated in Twitcher mice prior to onset of KD symptoms (27), and may be a promising alternative to support early treatment. However, further research is required to validate it for clinical use.

## Understanding KD Through Animal Models

KD is unusual among other rare diseases in having a range of animal models available that show reduced GALC activity. These animal models are listed by species and include cats, mice, dogs, sheep, and non-human primates, among which mice have been the most thoroughly characterized in terms of disease progression (Table 1).

**Table 1:** A summary of published studies characterizing various parameters in animal models of Krabbe disease.

**Animal model/mutation/mean survival of untreated animals**	**Parameter**	**References**
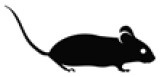 Murine (Twitcher) W339X 35-45 days	Weight	(28–30)
	Motor activity	(31–35)
	Psychosine levels	(29, 34, 36, 37)
	GALC activity	(34, 38, 39)
	GALC immunohistochemistry	(31–33)
	Myelination	(34, 39–41)
	Neuroinflammation	(32–34, 39)
	NMJ staining	(42)
	Neuroinflammation	(33, 34)
	Globoid cells	(33, 41, 43, 44)
	Immune cell counts	(29)
	Nerve Conduction	(30)
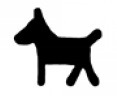 Canine (West Highland White Terrier) Y158S and P639S 16 weeks	Psychosine levels	(45, 46)
	Nerve Conduction	(45, 46)
	GALC levels	(45, 47, 48)
	Myelination; Globoid cells	(45)
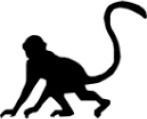 Non-human primate (Rhesus macaque) Δ387-388 (nuc) 1 year	Globoid cells	(49)
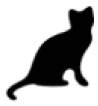 Feline (Domestic longhaired cat) Not known 21 weeks	Myelination (IHC); Globoid cells	(50)
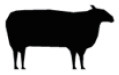 Ovine (Merino sheep) Not known Not known	GALC & Psychosine levels; Myelination; Globoid cells	(51)

Much of the current knowledge on KD was gleaned from characterization of the Twitcher mouse, which arose due to a spontaneous mutation (W339X) at Jackson Labs in 1976. The mouse and human GALC proteins bear 83% identity (7). Since its discovery, the Twitcher mouse has been well-characterized and used in a number of therapeutic studies. In general, the mice begin to show symptoms by 3 weeks, and live up to 35–45 days. Another model called the Twi-5j (with an E130K missense mutation in GALC) presents more aggressive symptoms and shorter life span compared to the Twitcher mouse, though reasons for this severity are not clear. Twi-5j mice begin to show symptoms by about 2 weeks and have a 21–30 day lifespan. These mice may have a stronger neuroinflammatory component than the Twitcher mice (52). The E130K mutation has also been reported in several patients with infantile KD (25). A third mouse model called twi-trs was generated by crossing the Twitcher mouse with a hybrid background mouse (129SVJ and FVB/N) bearing the same W339X mutation. These mice are larger in size and have a slightly extended lifespan of 50–62 days (53). Although the Twitcher mouse recapitulates the pathological symptoms of KD quite well, the symptoms are more severe in the peripheral nervous system. Unlike the Twitcher mouse, human pediatric KD patients with early onset disease generally present with more severe symptoms in the central nervous system.

A canine model of KD is available on the white terrier (Cairn and West Highland) background. The human and canine versions of GALC share 87% sequence similarity, though the 3' UTR in dogs is 1 kb shorter (54). Mutant dogs have two amino acid changes (Y158S and P639S), of which the former is implicated in causing disease (54). The model has been characterized in terms of its survival, body weight, GALC and psychosine levels, peripheral nerve conduction, brainstem evoked auditory responses, and neuromuscular strength. In general, the mutant dogs show disease symptoms by 6 weeks, and live up to about 16 weeks. Cerebellum and sciatic nerves appear to have the highest levels of psychosine accumulation (55). More recent reports indicate that psychosine levels are extremely high in the internal capsule of the cerebral hemispheres (45).

A rhesus macaque model with a two base deletion at positions 387–388 in exon 4 resulting in a frameshift in GALC and subsequent degradation of the resulting protein was first described in 1989 (56, 57). Clinically, these macaques are similar to humans and display significant weight loss, eating and respiratory difficulties, and muscle weakness. Severe demyelination can be seen in both the CNS and PNS of affected macaques, including both gliosis and the presence of multinucleated globoid cells (49). The amino acid sequence identity between non-human primate and human GALC is 97% (57). This model has been studied well, though not to the same extent as the murine and canine models. Other animal models that show reduced GALC activity like the merino sheep and the cat have not been well-characterized.

The common theme in all the animal models and human patients is the psychosine accumulation due to the reduction of functional GALC that results in disease symptoms associated with KD. Preclinical studies, largely centered on the Twitcher mouse, have demonstrated that disease symptoms can be reversed through various therapeutic efforts, indicating the appropriateness of these animal models for developing future therapies.

## Effects of Psychosine on Pathophysiology of KD

**Figure 4:**
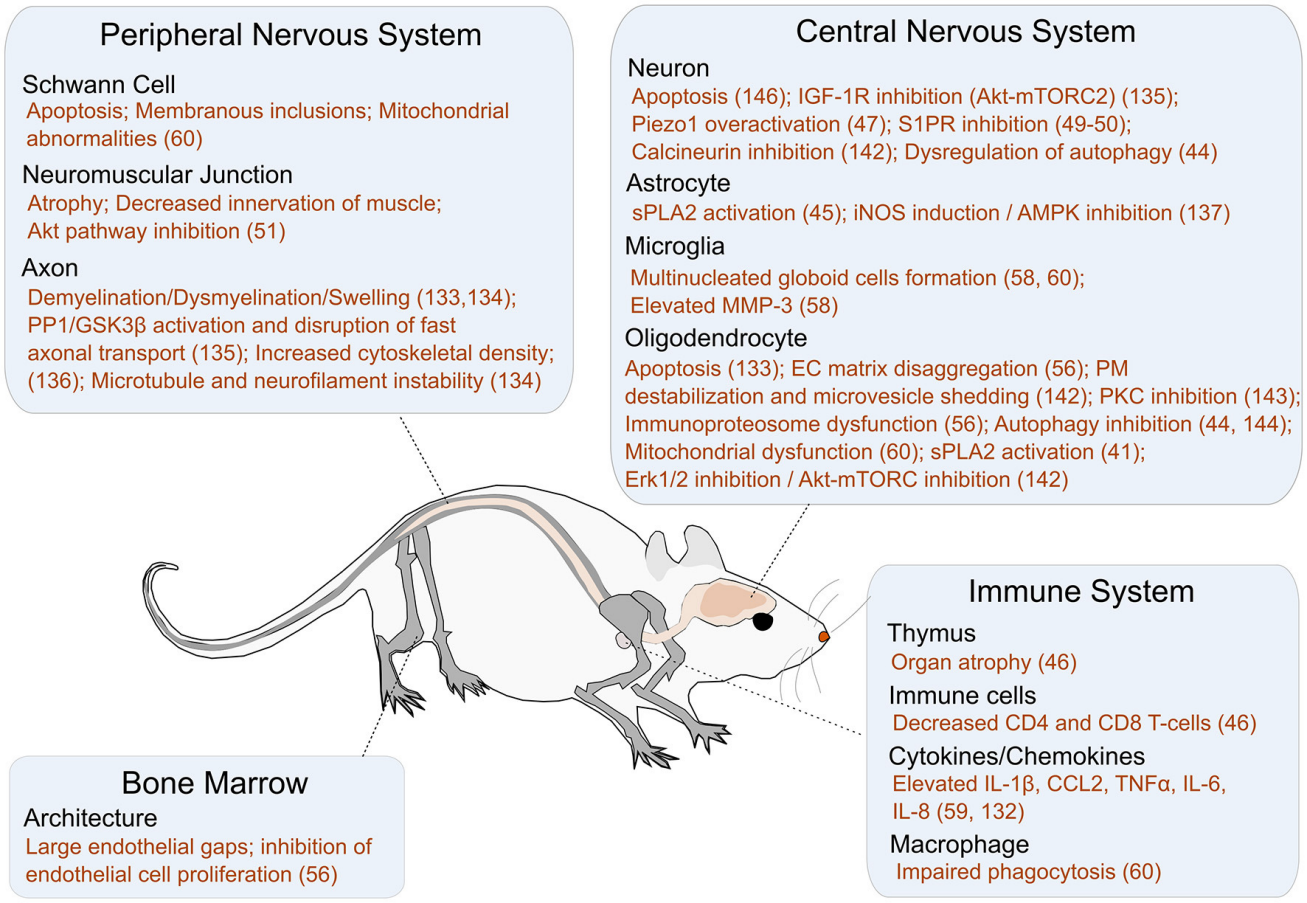
A summary of the impact of psychosine on different cellular targets and pathways. Additional references for this figure: Peripheral nervous system: Schwann Cells [Apoptosis, Membranous inclusions, and Mitochondrial abnormalities (61)], NMJ [Atrophy (62), Decreased innervation of muscle (62), and Akt pathway inhibition (62)], and Axon [Demyelination/Dysmyelination/Swelling (63, 64), GSK3B activation (42), Increased cytoskeletal density (65), Microtubule and neurofilament instability (64)]. Bone Marrow: Architecture [Large endothelial gaps (66), Inhibition of endothelial cell proliferation (66)]. Central Nervous System: Neuron [Apoptosis (63), IGF-1R inhibition (67), Piezo1 overactivation (68), S1PR inhibition (69, 70), Calcineurin inhibition (71), Dysregulation of autophagy (47)], Astrocyte [sPLA2 activation (72), iNOS induction (73), AMPK inhibition (73)], Microglia [Multinucleated globoid cell formation (61, 74), Elevated MMP3 (74)], and Oligodendrocyte [Apoptosis (75, 76), EC Matrix disaggregation (66), PM destabilization and microvesicle shedding (77), PKC inhibition (78), Immunoproteosome dysfunction (66), Autophagy inhibition (47, 79), Mitochondrial dysfunction (61), sPLA2 activation (80), erk 1/2 inhibition (77), Akt-mTORC inhibition (77)]. Immune System: Thymus [Organ atrophy (29)], Immune cells [Decreased CD4 and CD8 cells (29)], Cytokines/Chemokines [Elevated IL-1β, CCL2, TNFα, IL-6, IL-8 (81, 82)], and Macrophages [Impaired phagocytosis/Inflammatory phenotype (61)].

The psychosine hypothesis was proposed nearly 50 years ago and states that a loss in GALC activity leads to psychosine accumulation, which in turn causes the neural pathology seen in KD (58). The cellular function and concentration of psychosine at steady state remains to be determined. Given the variability in GALC activity levels and disease severity, particularly in late-onset KD patients, psychosine is used as a biomarker for KD diagnosis as well as for reliably distinguishing between early- and late-onset KD (59, 60). A summary of the effects of elevated psychosine levels is shown in Figure 4. We briefly describe recent findings elucidating the role of psychosine in several relevant areas including apoptotic pathways, demyelination, and neuroinflammation; details on the mechanistic role of psychosine in KD pathogenesis have recently been reviewed elsewhere (83).

### Apoptotic Pathways in Neural Cell Types

Oligodendrocytes and Schwann cells are particularly sensitive to psychosine-induced apoptosis during the initial stages of myelination and maturation (84). Although the precise mechanism is unknown, psychosine accumulation may increase lysophosphatidylcholine (LPC) and arachidonic acid levels in the cytoplasm, causing downstream inflammation and apoptosis in oligodendrocytes. Indeed, high levels of LPC have been observed both in the brains of human KD patients and Twitcher mice (80). In a recent study utilizing patient-derived iPSCs, a significant impact of GALC deficiency was observed particularly on the ability of progenitor cells to differentiate into oligodendrocytes at early timepoints (between day 7 and 14 following differentiation) (85). At least one important regulator of oligodendrocyte differentiation appears to be microRNA-219 (miR-219). Reduced expression of miR-219 in twitcher mouse oligodendrocyte precursor cells impeded oligodendrocyte maturation. Transfected miR-219 significantly reduced the number of apoptotic oligodendrocytes as well as psychosine levels in the differentiated cells (though not back to wild-type levels) (86). The mechanism of action for miR-219 in reducing psychosine levels is not yet clear. miR-219 also appeared to impact neuronal differentiation but the results were inconsistent between two independent iPSC lines. The differentiated neurons appeared to have an altered profile of lipids, suggesting deviations in lipid metabolism—an imbalance that could ultimately lead to apoptosis (85). Apoptosis in neurons may also proceed through psychosine-mediated ROS generation, as evidenced by both the presence of fundamental autophagy markers (LC3 and Beclin-1) and ubiquitin and p62 aggregates in the brain and sciatic nerve of both early and late symptomatic Twitcher mice (84). The p62 aggregates reduce the amount of autophagy receptors and cargoes and stall autophagy, leading to an accumulation of damaged proteins and organelles that easily oxidize and increase ROS levels. This study suggested that psychosine may be responsible for causing a dose and time-dependent accumulation of autophagosomes and autolysosomes in the CNS (47). In the peripheral nervous system, psychosine has been implicated in a caspase-3-dependent apoptosis through the Akt pathway (47). Interestingly, unlike in neurons, psychosine did not impair autophagy in astrocytes (47). However, a PLA2-mediated apoptosis was observed in astrocytes upon exogenous psychosine administration (72). These findings indicate that psychosine-mediated apoptosis in different cell types may proceed through different signaling pathways.

### Demyelination

Psychosine-mediated loss of oligodendrocytes and Schwann cells leads to widespread demyelination within both the CNS and PNS, and impacts non-neuronal organs as well. For example, demyelination in the spinal cord leads to thymic atrophy, with significant subsequent decreases in the number of CD4 and CD8 T cells in the Twitcher mice (29), though the clinical importance of this is unclear as the number of T lymphocytes in early or late onset KD in humans have not yet been investigated.

Recent research suggests that psychosine's impact on demyelination may also proceed through pathways that are independent of oligodendrocytes and Schwann cells. Specifically, two recent pharmacological studies expand our understanding of the potential mechanisms through which psychosine may induce demyelination by acting on neuronal membrane proteins. For example, in one study, GsMTx4, an agonist for Piezo1 (a mechanosensitive ion channel that regulates axon guidance and neural stem cell differentiation) reduced psychosine-induced demyelination in an *ex vivo* organotypic cerebellar slice culture model (68). This suggests that psychosine may contribute to demyelination by inhibiting Piezo1 function. However, additional research is needed to understand whether psychosine directly impacts Piezo1 function, and whether psychosine-induced demyelination could be reduced by upregulating Piezo1 function in an *in vivo* model. In a different study, an agonist of S1PR (G-protein coupled receptor that regulates both neuronal and glial cell function), fingolimod, was similarly found to reduce psychosine-induced demyelination in both organotypic cerebellar slice cultures as well as in Twitcher mice—though not to wild-type levels (69, 70, 87). The effectiveness of fingolimod in reducing psychosine-induced demyelination, while promising, is likely to have limited success as a therapeutic strategy because the available data is preventative at best, not therapeutic. Moreover, using fingolimod alone may have limited success, as the demyelination in KD appears to result from a concerted reduction in the activity of many receptors.

### Pathological Impact of Psychosine on Neuromuscular Junctions and Vasculature

Motor dysfunction and muscle weakness are common symptoms among KD patients that are also recapitulated in Twitcher mice, in which psychosine begins accumulating in the muscles soon after birth and increases over time (62). In the diseased mice, the soleus muscle neuromuscular junctions are significantly atrophied compared to wild-type mice. Twitcher mice appear to have both skeletal (gastrocnemius) muscle atrophy, decreasing innervation of the muscle, and reduced nerve-muscle contact area (88). Interestingly, the difference in the number of neuromuscular innervations between the Twitcher and wild-type mice was not significant (62). However, a 3D-imaging analysis of the microvascular architecture of Twitcher mouse cortex showed reduced density of microvessels, compared to wild-type mice suggesting that psychosine slows down angiogenesis (89).

### Role in Neuroinflammation

A comparative analysis of the Twitcher and wild-type mice nervous systems by Tandem Mass Tag Spectrometry based approaches indicated differences in more than 400 protein groups (90). Extracellular matrix proteins such as tenascin-C were elevated and the laminin meshwork were disaggregated in Twitcher mice, suggesting the presence of neuroinflammation (66, 91). Psychosine-induced oligodendrocyte death, aberrant signaling (involving PKC, CD200, or CD47) or even release of cytokines associated with oligodendrocyte stress (CCL2, IL-1B) may lead to microglial activation. Elevated levels of both CCL2 and IL-1B were detected in Twitcher mouse brains by qRT-PCR as early as postnatal day 2, likely due to increasing levels of psychosine (92). One of the defining features of Krabbe disease is the presence of multinucleated globoid cells. Interestingly, high levels of extracellular psychosine transformed cultured microglia into globoid cells, and this transformation was dependent on elevated levels of matrix metalloproteinase-3 (MMP-3) (74).

## Neuroinflammation and Globoid Cell Formation in KD

There is a growing appreciation for the role of dysfunctional microglia and astrocytes in demyelination in KD. As opposed to the psychosine hypothesis, the microglial hypothesis suggests a re-evaluation of the order in which the disease cascade occurs, namely that neuroinflammatory processes might precede disease symptoms and demyelination through the activation of astrocytes first, which then activate microglia in an abnormal manner (81). Post-mortem histology of the CNS showed that regions of demyelination frequently accompanied clusters of multinucleated globoid cells surrounding the axons. It is abundantly clear that aberrantly activated microglia and astrocytes contribute to inflammation through the release of cytokines and chemokines, such as CCL2, CCL3, CCL5, CXCL10, TNFα, IL-6, IL-1β, and IL-8, and recruit additional immune cells (monocytes and macrophages) from the periphery (81). Reduction in GALC function may result in pathology through pathways that are independent of psychosine or neuroinflammation and warrants further investigation. For example, GALC appears to be important in both early brainstem development as well as the maintenance of a stem cell niche in the bone marrow (61).

Globoid cells were first identified in Dr. Krabbe's report but their origin and contribution to KD pathophysiology are still unknown. These cells are frequently multinucleated, show periodic acid–Schiff (PAS)-positive staining, and are often observed early in the progression of the disease—even prior to demyelination or other symptoms. In conditional GALC knockout mice, macrophages (Ly6^high^/CD206^low^) assist in myelin degradation in the PNS. It is likely that the recruited GALC^−/−^ macrophages have impaired phagocytosis, accumulate lysosomes and myelin phagosomes and assume an inflammatory phenotype and a globoid appearance. Further analysis showed that treating bone marrow derived macrophages with galactosylceramide (C8-GalCer) but not psychosine recapitulated the phenotype *in vitro*. These induced globoid cells had reduced CD206 expression and elevated TNF-α and IL-1β cytokine expression profiles (61). A study selectively knocking out GALC in Schwann cells recapitulated the KD phenotype of fewer and smaller axons in the sciatic nerve of Twitcher mice (93). A similar approach could be used in better understanding the role of GALC-deficient microglia or astrocytes in disease pathology in the CNS.

Ceramide metabolism in the lysosomes may contribute to increased misfolding and aggregation of proteins (94). Oligodendrocytes from 6-day old Twitcher mice show functional defects in the immunoproteosome, as well as impaired protein synthesis and degradation (66). This study also reported an interesting connection with α synuclein, which has pathological associations with Parkinson's disease. This is particularly interesting in light of the fact that α synuclein aggregates have been found in Krabbe patient brain autopsies, and that psychosine binds to α synuclein to promote its aggregation (95, 96). Given these findings, it would be worth investigating the predisposition of patients with lysosomal storage disorders to other neurodegenerative diseases associated with protein aggregates (such as β amyloid plaques and tau neurofibrillary tangles). Lysosomal dysfunction might lead to impairments in synaptic transmission and ultimately neurodegeneration (97). Indeed, this is supported by recent GWAS studies that implicate lysosomal genes in the etiology of Alzheimer's disease, Parkinson's disease, and Frontotemporal Dementia (98–100).

## Therapeutic Strategies

Therapeutic approaches to treat KD symptoms over the past 20 years are described in the sections below.

### Hematopoietic Stem Cell Transplantation

Hematopoietic stem cell transplantation (HSCT) is currently the gold standard treatment for Krabbe disease. A seminal study in Twitcher mice demonstrated that HSCT administration following lethal irradiation in 10 day old mice could extend their survival from 40 to 80 days. Minor improvements were seen in gait, foraging, grooming, and body weight, but only when HSCT was administered at an early time point (101). In a subsequent study, GALC enzyme levels in various mouse tissues following HSCT administration were determined. In most tissues, GALC activity levels were in the normal range but in the CNS, GALC levels gradually increased over 2 months, ultimately reaching 15% of the CNS GALC levels in wild-type mice (102). Regardless, improvements in KD symptoms were apparent. These and other studies informed the use of HSCT in infantile KD patients, which can prolong life by several years if administered prior to the onset of symptoms.

In human KD patients, current clinical guidelines for applying HSCT differ for early and late onset KD. For early onset KD, HSCT is recommended in infants less than a month old who are asymptomatic. According to a recent study, although infants receiving HSCT prior to 31 days showed no significant difference in 5 and 10 year survival compared to infants receiving HSCT after 31 days, mobility and communication were in fact better in infants receiving HSCT earlier (103). In late onset KD, disease progression is first determined by monitoring symptoms over a 3–6 month time period. In addition, prospective recipients undergo complete physical and neurologic examination to determine their suitability for HSCT (104).

However, HSCT therapy does pose several challenges. One challenge with HSCT is finding a donor match for the patient. Patients receiving HSCT are at risk of an inflammatory response caused by the donor cells (graft vs. host disease). Another challenge is that for HSCT to be successful there needs to be presymptomatic intervention in the early onset KD patients. In this regard, both accurate diagnoses of children with KD as well as pre-symptomatic detection are important obstacles to be surmounted. One potential reason for the limited therapeutic efficacy of bone marrow transplantation seen in KD patients at symptomatic stages of the disease could be due to underlying defects in the BM vascular niche as a result of GALC deficiency (93). There is some evidence that functional BM vasculature may be essential for HSC engraftment (105). More recently, however, greater success has been observed in combining HSCT with other therapies, including gene therapies, which have previously been reviewed (106). There is some evidence that *de novo* GALC expression in hematopoietic stem cells is toxic (107). However, in one elegant experiment, miRNA expression (miR-126) specific to undifferentiated hematopoietic stem cells was identified and used to limit lentiviral GALC expression to progenitor cells (such as macrophages and microglia) alone. GALC-deficient mice infused with these lentivirus transduced hematopoietic stem cells survived for an additional 3–4 weeks, compared to untreated controls (108).

### Enzyme Replacement Therapies

Propelled by the success of enzyme replacement therapy in other lysosomal storage disorders including Fabry's disease and Gaucher's disease, a research group administered recombinant mouse GALC to Twitcher mice at day P10 intraperitoneally, with subsequent injections every other day (109). The observed minor improvements in survival (up to 1 week) and behavior could be due to the limited transport of the enzyme across the blood brain barrier (61). Delivering adequate levels of recombinant GALC across the blood brain barrier to achieve therapeutic efficacy is therefore a key obstacle for these enzyme replacement therapies. When recombinant GALC was administered intrathecally or intracerebroventricularly in Postnatal Day 2 (P2) Twitcher mice, treated mice survived up to 60 days, with significant motor function improvements compared to wildtype animals (110). In addition, the need for frequent administration of recombinant GALC due to its rate of turnover (1–2 days) also makes this approach less feasible for the clinic.

### Substrate Reduction

Reducing psychosine concentrations in tissues could be another strategy for ameliorating KD symptoms. One attempt used L-cycloserine, which deactivates serine palmitoyltransferase, an enzyme several steps upstream of the biochemical synthesis of psychosine (111, 112). A more recent approach utilized a β-cyclodextrin (cyclic oligosaccharide) to treat Twitcher mice, with the aim of absorbing excess psychosine, but the strategy showed limited improvements in overall survival—likely due to the molecule's inability to cross the blood brain barrier (113). A thorough review of substrate reduction therapies for KD has been published and is an excellent resource for a commentary on this therapeutic strategy (114). More recently, acid ceramidase inhibitors have showed significant reductions in brain psychosine levels in Twitcher mice and could be promising, particularly in combination with other therapeutic strategies (115). In general, however, substrate reduction therapies on their own have shown limited efficacy in preclinical models, and have not yet been tested in human KD patients, though they could be beneficial if used in conjunction with other approaches such as gene therapy.

### Viral Gene Therapy Initiatives

**Figure 5:**
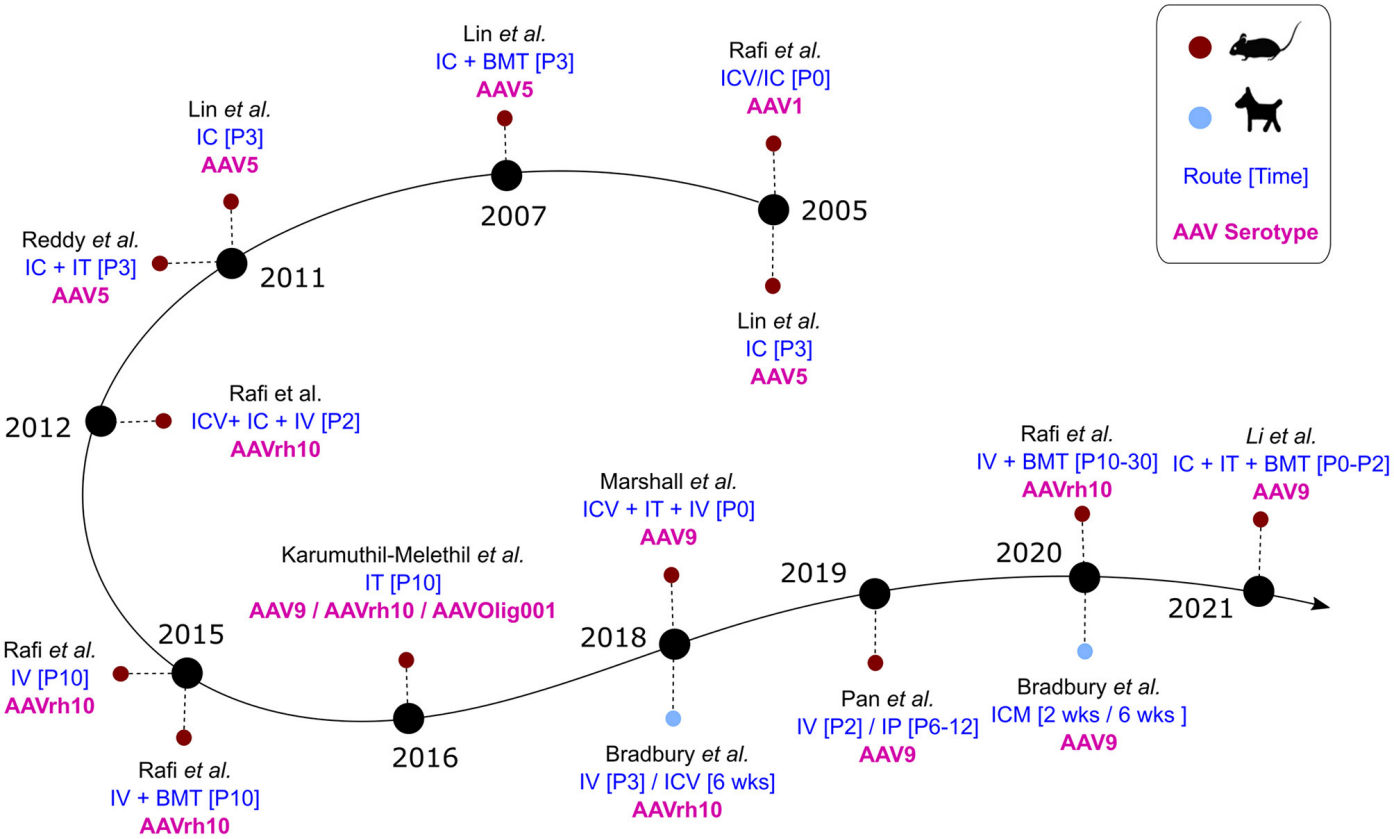
AAV studies in animal models of Krabbe disease over time. Blue: Route and time of administration. Pink: AAV serotype(s) used in the study.

Gene therapy approaches have been of particular interest to researchers working on Krabbe disease therapies due to the focus on the root cause of the disease. In addition to adeno-associated viral vectors (AAV), these approaches have also included adenoviral and retroviral vectors (116–118). In particular, a few different studies have investigated the efficacy of lentiviral (retroviral) delivery of GALC in patient iPSCs, Twitcher mice, and rhesus macaques (85, 118, 119). Lentiviral (LV) transduction of patient iPSCs successfully reduced psychosine accumulation and provided a partial rescue in differentiation—with greater improvement observed in neurons than in oligodendrocytes (85). Similarly, two studies investigated intracerebral injection of LV in Twitcher mice, and showed effective production of GALC from neurons, astrocytes, and oligodenderocytes. However, improvement in either motor skills or life span (extension of ~2 weeks) was limited (118, 120). LV GALC was also investigated in the rhesus macaque model of Krabbe disease—the first time gene therapy for KD was attempted in the NHP model. As in mice, the lentivirus efficiently transduced neurons, astrocytes, and oligodendrocytes, and GALC levels in the diseased monkey were restored to near physiological levels (119). Of note, the LV-treated diseased monkey showed significant improvement in neuromuscular strength within 3 months post therapy—with scores comparable to age-matched normal animals. Unfortunately, limited information can be drawn from this study, as only one NHP each (affected or control) was used. Compared to AAVs, LVs are able to package transgenes up to 10 kb, which is twice the length achievable with AAVs. In particular, for *ex vivo* gene therapy strategies in which long-term transduction of hematopoieitic stem cells is needed, LVs are recognized as better vectors than AAVs, which are primarily non-integrative unless combined with genome integration approaches (121). However, in the context of *in vivo* approaches, LVs appear to have limited capacity for diffusion post-inoculation due to their larger size compared with AAVs and bear significant risk of insertional mutagenesis. As of this review, 15 studies have been published since 2005 using AAVs to deliver GALC in animal models (Figure 5). Of the 15, two studies were done on the canine model, one was done in twi-trs Twitcher mice, and the remaining 12 were done in Twitcher mice. In addition, a recent announcement by PassageBio using the canine model has indicated a positive outlook in the development of an AAV-based therapeutic construct that rescues GALC expression (122). While the goal of all these studies was to restore GALC to physiological levels, they differed in the AAV serotype and dosage, as well as the route and time of administration. Importantly, some of these studies investigated the combination of AAV administration with bone marrow transplantation (BMT) in prolonging therapeutic efficacy.

### AAV Serotype, Dose, and Route of Administration

Two groups led the way in exploring AAV-based treatment for Krabbe disease, highlighting the importance of AAV serotype in ameliorating symptoms (28, 53). The main endpoint criteria for evaluating the efficacy of these studies was the median survival of the treated mice, though later studies also included measures of neuromuscular function and strength.

Administration of AAV1 at a total dose of 3.0e13 vgs/kg in the brain parenchyma and ventricles (IC + ICV; 1.5e13 vgs/kg for each site) increased the lifespan of Twitcher mice by 2–3 weeks, showing high GALC expression in the hippocampus and olfactory bulb but undetectable levels in the cerebellum (53). The mice showed numerous globoid cells as well as lack of myelin regeneration, with limited GALC expression in oligodendrocytes and astrocytes. These results highlight the importance of restoring GALC expression across cell types and potential limitations of restoring sufficient GALC activity through cross-correction, the process by which GALC-deficient cells can uptake secreted GALC from the extracellular environment. Intracranial (IC) administration of 7.2e12 vgs/kg by AAV5 also increased the lifespan of Twitcher mice by only 2 weeks, despite more widespread GALC expression (28). In addition to the hippocampus, GALC was expressed in the cerebral cortex, thalamus, and choroid plexus. While behavioral data is not available from the AAV1 (IC + ICV) study, AAV5 (IC) treated mice showed minor improvements in neuromuscular coordination (based on their performance on rotarod) and no improvement in neuromuscular strength (based on the wire hang test data). These findings indicated the limited therapeutic effect of a localized CNS delivery of GALC on lifespan and neuromuscular function.

Two follow-up studies published in 2011 by the Sands group used AAV5 in Twitcher mice, though the route of administration for each was different. AAV5 was administered either IC alone at a dose of approximately 7.20e12 vgs/kg (with a specific focus on delivering the virus to the hippocampus, cerebellum, and the neocortex) (32) or it was administered IC in combination with an intrathecal (IT) injection (a combined dose of 1.76e13 vgs/kg) (33). The rationale behind cerebellar administration was to allow GALC to disseminate to the midbrain, the brainstem, and spinal cord. In the IC only study, there was significant gliosis in the spinal cord that also corresponded to 6% of wild-type GALC levels (32). The median lifespan increased by only 3 weeks, however marked improvements were observed in weight gain and neuromuscular strength. In the second study (IC+IT), the intracranial injection focused on viral delivery to the forebrain and cerebellum, along with an injection to the spinal cord at 7.8e12 vgs/kg and 9.75e12 vgs/kg, respectively (33). Broad recovery of GALC across the CNS resulted in reduced levels of microgliosis, astrogliosis, and psychosine accumulation in both the brain and spinal cord, and translated to an increase in the median lifespan of treated mice by 4 weeks (the longest observed survival at the time), and significant improvements in neuromuscular coordination. However, no improvement could be observed in the neuromuscular strength of the treated mice, indicating that restoring GALC function in both the CNS and PNS are needed for long-term therapeutic efficacy.

From 2012 to 2020, seven different studies used either AAVrh10 or AAV9 in Twitcher mice as therapeutic preclinical efforts for KD (Figure 5). One of these studies used IT delivery of AAV9 (self-complementary or single stranded), AAVrh10, and AAV-Olig001 (specific for oligodendrocytes), each at 4e13 vgs/kg, and was largely unsuccessful in extending lifespan in the treated mice beyond 2 weeks (40). The remaining studies were either intravenous only or intravenous combined with other routes at doses ranging from 5.3e12 to 4.0e14 vgs/kg (Table 2). It seems that the doses used were suboptimal in achieving effective levels of GALC across the blood brain barrier.

**Table 2:** A summary of published AAV therapies in animal models of Krabbe disease with publication year, AAV serotype used, promoter element and other modifications, route and time of administration, and median survival in days.

**Publication**	**Year**	**AAV vector**	**Promoter**	**Other modifications**	**Route (day of injection)**	**Survival (days)**
Rafi et al.	2005	AAV1	CMV		ICV + IC (P0)	55
Lin et al.	2005	AAV5	CBA		IC (P3)	52
Lin et al.	2007	AAV5	CBA		IC / + BMT (P3)	49; 104
Lin et al.	2011	AAV5	CBA		IC (P3)	62
Reddy et al.	2011	AAV5	CBA		IC + IT (P2-P3) / + BMT	71; 123
Rafi et al.	2012	AAVrh10	CBA		ICV + IC + IV (P2)	104
Rafi et al.	2015	AAVrh10	CBA		IV (P10)	75
Rafi et al.	2015	AAVrh10	CBA		IV + BMT (P10)	100–350
Karumuthil-Melethil et al.	2016	AAV9; AAVrh10; AAV-Olig001	CBA / CBA / minJET	Codon optimization	IT (P10)	50–55
Marshall et al.	2018	AAV9	CBA	Codon optimization	ICV + IT + IV (P0-P1)	263
*Bradbury et al.	2018	AAVrh10	CBA		IV (P3) + ICV (P42)	300
Pan et al.	2019	AAV9	CAG	IDS Signal Peptide; hApoB-LDLR-BD	IV (P2); IP (P6-12)	150; 104
Rafi et al.	2020	AAVrh10	CBA		IV + BMT (P10-40)	450–500
*Bradbury et al.	2020	AAV9	CAG	Codon optimization	ICM (P14 / P42)	900+
Li et al.	2021	AAV9	CBA		IC + IT / + BMT (P0-P2)	250–300

The asterisk (*) marks the studies done in the canine model of Krabbe disease; the rest of the studies were done using the Twitcher mouse. Blank spaces denote information not available in the public domain. BMT, Bone marrow transplantation; IC, intracranial; IT, intrathecal; ICV, intracerebroventricular; IV, intravenous; IP, intraperitoneal; ICM, intracisternal.

The Wenger laboratory published two studies in Twitcher mice using AAVrh10 administered either as a combination of IC, ICV, and intravascular (IV) injection or IV alone (39, 123). In the first of the two studies (IC+ICV+IV, total dose of 5.3e12 vgs/kg; 7.5e11 vgs/kg each for ICV and IC, and 3.8e12 vgs/kg for IV), the median survival in treated mice increased by 8 weeks and the treated mice performed comparable to wild-type on several behavioral parameters within the first 4 weeks of treatment (123). Over the next 3 months the treated mice showed significant decrease in vertical activity indicating growing weakness of the hind limbs over time. Pathological markers such as multinucleated globoid cells, though absent initially, began to appear in the cerebellum, spinal cord, and sciatic nerve by P140. Demyelination was also seen in the cerebrum and brainstem. The IV alone treatment at 4.0e13 vgs/kg did not have a significant impact on extending lifespan but the treated mice showed normal neuromuscular coordination (based on gait pattern). These studies highlight the importance of having sustained benefits to the motor functions (124). While intravenous administration of AAV appeared to have a clear benefit in significantly improving neuromuscular function, these studies indicated that achieving a balance between dose and route of delivery would be critical for long-term therapeutic benefit.

Two additional studies administered AAV9 to Twitcher mice either IV in combination with ICV and IT (34), or IV alone (35). The latter of the two studies (IV alone; 4e14 vgs/kg), showed an average increase in survival of 16 weeks in the treated Twitcher mice. In this study, hind leg dragging, a characteristic phenotype of untreated Twitcher mice, was significantly delayed in the treated mice by 8–10 weeks. The other study [ICT (4.5e12 vgs/kg) +IT (4.13e13 vgs/kg) +IV (1.65e14 vgs/kg), total 2.1e14 vgs/kg] extended the lifespan of Twitcher mice by an average of 32 weeks. This is the longest that any stand-alone AAV therapy has yet been able to accomplish in extending lifespan in the Twitcher mice. Moreover, the neuromuscular strength of the treated mice seemed comparable to wild-type mice. These results were reflected in the low levels of psychosine in the brain, spinal cord, and sciatic nerve of treated mice at P40. However, at the terminal timepoint, levels of psychosine in each of these regions was significantly high (34), likely indicative of decreasing GALC levels due to episomal dilution in proliferating oligodendrocyte progenitor cells and glia over time (125).

Finally, most recently, two studies in the canine model of KD (45, 55) attempted to rescue GALC expression in both the CNS and PNS. In the first of the two studies, AAVrh10-cGALC was distributed across both IV and ICV routes, divided evenly, at either a high (3.8e13 vgs) or low dose (1.2e12 vgs) (55). In this study, the lifespan of treated dogs was extended by an average of 21 weeks, with one dog surviving up to 43 weeks, an extension of 27 weeks. Although at a terminal timepoint, these dogs showed normal nerve conduction velocities in the tibial, sciatic, ulnar, and radial sensory nerves, the animals still developed a pelvic limb spastic paralysis—likely reflecting an increase in psychosine levels over time. This study also reported improvements in demyelination and reductions both in the number of globoid cells and neuroinflammation (gliosis). However, significant demyelination was observed in the spinal cord, suggesting incomplete rescue of GALC in the CNS (55). In the second study, AAV9 cGALC was administered IT into the cisterna magna. Although IT administration previously in the Twitcher mice had not been successful, the approach did have several advantages including higher transduction of the CNS, immune-evasion, and the use of less vector. This study reported great success in prolonging the life of the animals to beyond 2.5 years of age, with the animals still surviving at the time of publication of this study. At 16 weeks, these dogs showed global GALC expression, significant reduction in psychosine levels in both the CNS and PNS, and restoration of both myelination as and nerve conduction velocities (45).

The importance of using the right dose was recently demonstrated in three different studies (one in Twitcher mice, two in dogs). In the Twitcher study, 1 day post-BMT treatment, Twitcher mice were injected IV with different doses of AAVrh10 (4X, 1X, 1/2X, 1/10X, and 1/100X). The 1X dose of AAVrh10 was equivalent to 4e13 vgs/kg, and a dose-dependent increase in lifespan was reported, with the exception of the 4X dose (126). Interestingly, there appeared to be no significant difference (in lifespan) between the 1X and 4X dose, indicating that the levels of GALC with the 1X dose might already have reached saturation. Similarly, in the first of the two canine studies, the animals treated with the low dose of AAV (1.2e12 vgs) showed only minor improvements in disease symptoms. In contrast, the animals treated with the high dose of AAV (3.8e13 vgs) not only had fewer symptoms of KD, but also lived much longer (55). Finally, in the second of the two canine studies, a higher dose of AAV9 (1e14 vgs/kg) delivered ICM elicited the maximal therapeutic benefit in dogs, extending survival in treated dogs to beyond 2.5 years which is 8x more than untreated dogs (45). The low dose cohort (2e13 vgs/kg), in contrast, did not survive as long (up to 35 weeks of age) and developed an attenuated form of KD which included blindness and motor defects prior to death.

As a final note, the brain regions and neural cells transduced by AAVs depend not only on the serotype of AAV used, but also on additional variables such as promoter elements in the cassette and route by which the AAV is administered. For instance, in *in vitro* cultures, AAV1 and AAV9 both strongly transduce neurons; however, *in vivo*, following brain injections, AAV1 transduction of neurons in the cortex is low when compared to transduction using AAV9 (127). Similarly, when administered intravenously, AAV9 can penetrate the blood brain barrier, whereas AAV1 cannot. Furthermore, the cell tropism of AAV9 when administered intravenously is different than when administered directly into the brain. Extensive discussions on targeting AAVs to specific brain regions and neural cell types by leveraging these parameters have previously been reviewed (127–130). However, we would like to note that all the AAV constructs described in this review that showed therapeutic efficacy utilized ubiquitous promoters like the chicken beta-actin (CBA) promoter or CAG promoter to express the GALC transgene, indicating the need for widespread expression of GALC to achieve therapeutic efficacy.

### Time of Administration

A general approach in the treatment of CNS-based diseases is to treat patients as early as possible to prevent potential irreversible pathological changes in the nervous system. HSCT in early onset KD patients appears to be most successful when administered pre-symptomatically. It is likely that infiltration of GALC and subsequent cross-correction in the CNS requires several weeks though the exact timing for the process is not clear.

One study explored the possibility of administering 4.0e13vgs/kg of AAVrh10 to P10 mice through an IV tail vein injection (39). The myelination status of the P10 mouse is considered to be similar to a newborn baby (131). At P60, 18 of the 20 treated mice showed GALC levels in the CNS comparable to that of wild-type mice. However, there still was evidence of pathology including globoid cells in the brain and smaller size of the myelinated axons in the sciatic nerve. The overall survival in the P10 mice, compared to P2 mice, was greater by 1–2 weeks and it is plausible that this was because of the significantly greater dose rather than timing of administration (124).

Two additional studies evaluated administration of AAV9 to Twitcher mice either at P0 (IV+IC+IT, 1.1e14 vgs/kg) or P2 (IV, 4.0e14 vgs/kg) (34, 35). Both of these studies reported significant major improvements in lifespan and sustained neuromuscular function. In another recent study, AAVrh10 (IV, 1.0e14 vgs/kg) was administered to Twitcher mice at various time points following BMT, including P11, P15, P20, and P40 (126). While administering AAV at P40 did not result in an increase in lifespan compared to BMT treatment alone, a significant increase in lifespan could be seen if AAV was administered at P11, P15, or P20 instead. However, among the P11, P15, and P20 treated Twitcher mice, there was no significant difference in lifespan extension, and the sciatic nerve of the P20 group in particular was inadequately myelinated. This is in line with evidence showing that after P15, the skeletal muscle architecture begins to be significantly impaired (88), suggesting that there is indeed a therapeutic window within which the maximum benefit of AAV-based gene therapy can be realized. Unfortunately, no data was included in the AAV study to indicate the status of re-myelination in the CNS for any of the timepoints, nor any behavioral tests reported that could provide additional insight into the potential impact of the time of AAV administration on neuromuscular function. Therefore, it is difficult to definitively state whether the time of administration affected any parameters other than lifespan and myelination.

Two studies by Bradbury et al. in the canine model also indicate that the earlier the intervention, the better the outcome (45, 55). In both studies, mutant dogs that showed highest survival were treated prior to disease onset (6 weeks). In the first study, the animals were treated with AAVrh10-cGALC on Day 3, and in the second study, the animals received therapy at 2 and 6 weeks of age (45, 55). Although the canines treated at this later time point did not survive as long and developed pelvic limb paralysis, they did show nerve conduction velocities restored to near normal and a significant slowing down of disease progression. This suggests that post-symptomatic treatment of human KD patients using gene therapy could still be beneficial.

### Combining AAV With BMT

HSCT has been established as the “standard of care” for pre-symptomatic Krabbe patients because it showed the most effectiveness in slowing the course of the disease and preserving cognitive function (132). Patients do however show peripheral neuropathy after a decade and to address this, combining HSCT with another therapeutic strategy could be beneficial to patients. HSCT treatment supplants GALC-expressing myeloid progenitors that combine the removal/substitution of GALC-deficient myeloid progenitor cells with the therapeutic effects of cross-correction which is not possible with AAVs. A report has shown that HSCT treatment in twi mice lowers psychosine levels in the sciatic nerves effectively. One drawback was that though there was initial robust lowering of psychosine (up to 70%), the effects were not sustained, and the mice died soon after they reached 100 days (38). A combination of AAV and BMT attempted by the Wenger lab showed robust GALC expression in the sciatic nerve and spinal cord when the diseased mice were treated with various doses of AAV with BMT (126). Interestingly, in the absence of a BMT alone control, although we do observe increasing expression of GALC depending on the AAV dose, it is difficult to tease out the contribution of BMT in alleviating the peripheral neuropathy (126). This study reported both a significant increase in the lifespan of the mice (up to 700 days) and efficient myelination of the sciatic nerve (126). Furthermore, preclinical studies with AAV5/BMT treatment had a more complete and widespread reduction of both GFAP+ and CD11b+ cells than either the AAV5-treated or BMT-treated animals alone and was able to extend the average life span of the Twitcher mice to 103 days (range = 38–153 days), that was considerably longer than any previous therapeutic approach (33). These data suggest that a combination of HSCT with AAV-based gene therapy could address the shortcomings of either approach used separately. However, a combination approach would come with its own set of cautionary notes.

As one example, results of an AAV/BMT combination therapy in the murine MSP IIIB model indicate that a combined approach does not always result in synergistically improved efficacy. This study surprisingly demonstrated even lower efficacy in the combination therapy treated group than the AAV alone treatment, presumably due to antagonistic effects of the two approaches (133). In addition to such effects, there are logistical challenges associated with pursuing a combined approach in clinical trials. Importantly, not only would an AAV/BMT combination therapy approach dictate selection of patients with low titers of neutralizing antibodies to AAV capsids (for effective AAV transduction), but also would require finding donor matches for such patients. Notwithstanding these, the AAV-BMT combination approach is currently being utilized in the RESKUE clinical trial conducted by Forge Biologics (NCT04693598). Emerging data from this study will help determine the clinical efficacy of such an approach.

Given that HSCT/BMT is the current gold standard for treating Krabbe disease, nearly half of all the AAV studies investigated the combined efficacy of AAV and BMT in prolonging survival in Twitcher mice. Studies combining AAV with BMT can be difficult to interpret in terms of the observed longevity and overall improvements due to the inherent variability in BMT-mediated engraftment. BMT involves the use of radiation or chemotherapy to eliminate myeloid cells, and depending on the intensity of radiation used (myeloablative or myeloreductive conditioning) or the choice of chemotherapeutic agent (such as busulfan or cyclophosphamide), may cause unintended cellular changes. For instance, Reddy et al. (33) reported that even with low-radiation myeloreductive conditioning, two of the three Twitcher mice in the AAV+BMT group had cerebellar dysplasia and had lost much of their normal cerebellar architecture. This was not true for the AAV-only group, indicating that the radiation used in the procedure may have undesirable effects.

Combining AAV5 (IC) and BMT (myeloreductive) showed a profound effect on multiple aspects of KD in Twitcher mice (134). The regimen for myeloreduction in this study involved a single conditioning dose of 400 rad from a ^137^Cs source; this was followed by an IV injection of unfractionated bone marrow suspension. The combination therapy in this study extended the lifespan in Twitcher mice by an additional 50 days (than with either therapy alone). Additionally, there was decreased astrogliosis, the mice seemed to maintain their weight longer, and showed marked improvement in neuromuscular coordination. Less improvement was observed in neuromuscular strength despite the combined therapy. It is likely that BMT was either providing supplemental GALC for cross-correction (where AAV was unable to provide it, such as in the periphery) or was reducing overall neuroinflammation. Another study showed that BMT without any immunosuppressive preconditioning had a negligible effect on GALC activity or psychosine levels in the brain or spinal cord (34). An important consideration here is that the data was obtained a month after BMT, and infused cells could take longer to infiltrate into the CNS. It is plausible that while AAV therapy provides an immediate source of GALC, BMT provides a steady but insufficient source of GALC. Indeed, psychosine levels in the CNS of mice treated only with BMT did not seem to be impacted at P40, but were greatly decreased at terminal endpoints (135), indicating that BMT-delivered GALC takes much longer to be effective in the CNS. Animals in the AAV+BMT group initially showed better neuromuscular coordination compared to AAV alone. With AAV supplying GALC in the CNS, BMT likely provided GALC to the PNS for cross-correction as well as helping with myelin turnover. However, since the amount of cross-correction that BMT is providing in the PNS is likely minor (in the absence of AAV-driven GALC expression in the PNS), psychosine levels in the sciatic nerve accumulate over time beyond a threshold level, at which point the BMT alone is not enough to correct PNS-driven pathology.

Rafi et al. (39, 124) combined BMT (with busulfan-mediated myelosuppression) and the systemic administration of AAVrh10 at P10. In this study, the combination of AAV and BMT extended the lifespan of several of the treated mice to >300 days. A sustained rescue was noted in the gait, hanging and grip strength assays in treated mice. In this study, it is likely that the AAVrh10 provided supraphysiological levels of GALC to the PNS (39). Indeed, the significance of AAVrh10-delivered GALC in re-myelination of the sciatic nerve can be seen in a study in which BMT was combined with an IT-only administration of AAVrh10 (40). In this study, GALC levels in the sciatic nerve were not sufficient, and compared to heterozygous controls, only partial rescue of axonal loss and re-myelination could be observed. Finally, in a recent study, the dose-dependent relationship of systemic AAVrh10 administration and sciatic nerve re-myelination was demonstrated (126).

### Limitations of AAV Gene Therapy

Despite their promise, however, AAVs as vectors for gene therapy are not without their limitations. A key limitation of using AAVs is defining the dose that is both safe and efficacious in human patients, particularly when comparable doses in preclinical models do not show significant side effects. This limitation is particularly relevant for a disease such as Krabbe disease, where high levels of GALC seem to be critical in extending survival as well as neuromuscular function. Furthermore, even if high levels of GALC are achieved at a clinically tolerable dose, it is not known whether AAV-mediated GALC overexpression could result in gain of toxic function. Long term AAV9-mediated overexpression of survival motor neuron (SMN) protein, a protein associated with spinal muscular atrophy, was recently shown to cause toxicity in the sensorimotor circuit in mice (136).

Although less ideal than a single-dose therapeutic, one could envision maintaining adequate levels of GALC across both the CNS and PNS through recurring AAV injections. However, another limitation of using AAVs is that multiple injections of the same virus over time are not possible due to neutralizing antibodies. Nevertheless, the use of substantially different clades of AAVs to evade the immune response could still be a viable strategy. Additional strategies for evading the immune response include using decoy empty capsids to saturate circulating neutralizing antibodies, generating novel viral capsids through directed evolution, and chemically modifying viral capsids (137, 138). Furthermore, if the on-going efforts in engineering AAVs that can evade the immune system are successful, these approaches would be worth considering.

Although rAAVs have traditionally been categorized as relatively non-immunogenic, two recent studies have reported the occurrence of neuroinflammation in the dorsal root ganglia of the spinal cord and an acute syndrome of thrombocytopenia, hepatic and renal toxicity (139, 140). Both studies implicated a high dose of AAV either by intrathecal or intravenous administration. Recent data from the ASPIRO trial (NCT03199469) for X-linked myotubular myopathy (XLMTM) reported two patient deaths from sepsis. The experimental gene therapy AT-132 that expresses the therapeutic MTM1 payload was packaged in an AAV8 capsid that has shown an excellent safety profile in previous clinical profiles. Relatedly, several other severe adverse events (of varying severity) have occurred in patients receiving high dose systemic AAV largely stemming from innate immune responses (e.g., inflammation and complement activation) and cellular immune responses to vector days-to-weeks following intravenous administration. It is possible that the adverse events arose due to the high dose of 3 × 10^14^ vector genomes per kilogram bodyweight (vg/kg) administered to the patients. Assuming the patient body weight to be around 30 lbs, they would have received a dose in excess of 4 × 10^15^ vgs. Compounding this is the fact that the patients had pre-existing hepatobiliary disease that could have exacerbated toxicity given that most AAVs traffic to the liver irrespective of intended target organ or route of delivery. This data strongly directs setting an upper limit on intrathecal and intravenous AAV dosing though it is still unknown if anti-inflammatory or immune suppressive medications would be helpful in mitigation. In the absence of detailed reports on the clinical trials, it is imperative to proceed with caution when extremely high doses are being considered for therapy.

Additionally, AAV gene therapy in the murine MPS VII model was implicated in hepatocellular carcinoma (HCC) as the malignant cells showed integrated AAV sequences (141). Logan et al. have suggested that a liver-specific enhancer-promoter region in the 3′ untranslated region (UTR) could be linked to developing HCC risk (142). It is worth noting that although this region (a stretch of 124 nucleotides near the 3′ ITR) is within the non-protein-coding sequence of the wild-type AAV2 genome, part of this sequence may be present in recombinant AAV genomes currently being used for gene therapy. Similarly, Chandler et al. demonstrated that the overexpression of proximal microRNAs and retrotransposon-like 1 gene (Rtl1), following AAV integration into the RNA imprinted and accumulated in nucleus (Rian) locus, was associated with HCC (143). In a separate study using Twitcher mice with HCC tumors integrated in the Rian locus, it was suggested that the radiation and L-cycloserine treatments may have contributed to the development of HCC following AAV gene therapy (144). Yet another AAV editing vector targeting the Rian locus produced HCC in all injected mice, though it is important to note that animals treated with the control vector developed HCC as well, albeit at a lower frequency (145). Though the Rian locus is not present in humans, upregulation of delta-like homolog 1-deiodinase type 3 (DLK1-DIO3) locus, the human ortholog of the Rian microRNA locus, has been associated with a poor survival rate in patients with HCC (143). Long-term studies in a higher species namely dogs with Hemophilia A showed clonal expansion of cells that harbored integrated vectors indicating potential for genotoxicity in AAV treated animals (146).

Similarly, clonal insertion of wild-type AAV2 genome sequences in human HCC liver biopsies have also raised concerns about the potential genotoxicity of AAV based gene therapy (147). One important aspect of this study was that the identified AAV sequences were mostly fragments of the ITR integrated in HCC pro-oncogenes which were possibly subject to the ITR transactivation effect. These observations were recently corroborated by the identification of liver-specific enhancer-promoter elements in the wild-type AAV2 genome. These elements (present in a stretch of 124 nt) were found to be close to the 3' ITR and within the 163-nt common insertion region of the AAV genome, which was previously identified in HCC biopsies (142). Therefore, AAV vector transgenes devoid of this liver trans-activating genome region would be preferred for clinical use compared with those containing it. Finally, in a recent study, single-molecule real-time DNA sequencing was used to investigate chromosomal rAAV integrations in primary human hepatocytes both *ex vivo* as well as *in vivo* (using a humanized liver mouse model) (145). This study, involving multiple rounds of replication post-AAV transduction, found 0.6–2.8% of hepatocytes to have stably integrated the reporter transgene, and validated the earlier finding that a majority of the integrations were associated with the 3' ITR. As our ability to sequence both concatameric rAAV integrations and GC-rich regions improves, our understanding of the genotoxic risk of using AAV vectors *ex vivo* or *in vivo* will broaden. In addition, how chromosomal integrations vary with AAV serotype, therapeutic dose, and cell type (such as how frequently a cell divides) are all variables that require further investigation.

In conclusion, we would like to highlight the elegant argument that WT AAV insertions in hepatocellular carcinoma do not inform debate over genotoxicity risk of vectorized AAV (148). Interestingly a study also showed that AAV vector dose, enhancer/promoter selection, and the timing of gene delivery were all critical factors for determining HCC incidence after AAV gene delivery indicating careful vector and study designs would potentially be able to address toxicity concerns (143). It is also important to note that some lethal pediatric disorders that arise from inborn errors of metabolism may not have any other recourse and that the risk to benefit ratio of toxicity and therapy would need to be balanced to provide lifesaving therapeutics to patients.

## Discussion

Given the broad expression of GALC in the nervous system, a therapeutic that rescues GALC locally is likely to have limited success. This hypothesis is borne out by multiple studies where localized GALC rescue failed to improve neuromuscular function (28, 31, 53). At least in mice, it has been reported that introduction of sufficient levels of GALC as broadly and early as possible to the brain, the spinal cord, and the sciatic nerve appears to be essential for long-term development. Currently, HSCT is the gold standard for treating Krabbe disease, and if administered to infants prior to onset of symptoms, significantly improves patient quality of life. However, despite these significant improvements, it has been observed that peripheral neuropathy in HSCT-treated patients continues to worsen (149). This suggests that the GALC rescue, and the relatively slower rate of enzymatic cross-correction, provided by this approach is inadequate for long-term therapeutic efficacy. Therefore, a gene therapy based approach for treating Krabbe disease holds great promise. This is substantiated by the significant improvements in lifespan and neuromuscular function observed for mice treated with broadly transducing AAV serotypes such as AAV9 and AAVrh10 (34, 35, 124, 126). Learnings from the studies reviewed here provide insight into how we may design a successful therapeutic for Krabbe disease.

In mice, myelination in the spinal cord begins at birth and continues till P60, therefore delaying treatment would allow psychosine to begin impacting early development of cells in the PNS (150). In support of this, the induced deletion of GALC prior to P4 in a conditional knockout mouse model resulted in severe neurodevelopmental defects (61). This study also revealed that at P5, the expression of GALC was highest in immature T-box-brain-1 positive brainstem neurons. T-box-brain protein 1 is a neuronal transcription factor that is involved in numerous developmental pathways including neuronal migration, axonal projection, and cortical development. Expression of GALC within these neurons indicates that GALC could also be playing a critical, yet currently unknown, role in the development and function of brainstem neurons. This may be the reason why KD progression in Twitcher mice was not impacted following administration of AAV through ICV and IC at P7 (instead of P0), and no increase in lifespan of treated mice was observed (53). The importance of GALC in early developmental stages may also be gleaned from improvements observed in weight gain and neuromuscular strength when GALC rescue was observed in Purkinje neurons (32). Purkinje neurons are critical in maintaining the correct polarity and cytoskeletal arrangement of neuronal cells in the cerebellum, and dysfunction within these cells can lead to interrupted neuronal circuitry between the brain and spinal cord. It would also be prudent to pay particular attention to the time of administration, especially in the context of systemic administration of AAV therapies. This is because with age, systemically administered AAV might face greater obstacles in its ability to transduce different parts of the CNS due to the blood brain barrier. For instance, following IV inoculation of AAVrh10 in Twitcher mice at P11, it took at least 4 days before GALC activity in the brain of the treated mice could match wild-type levels (126). This is likely due to the age of mice (P11), and following AAV delivery, psychosine may be still be accumulating unchecked for an additional 4 days. Whether this time would be shorter following IV administration in a P2 mouse is not yet clear, and a comparison of the time it takes for GALC levels to reach wild-type levels in P2 mice following IV administration could provide additional insight. This may also be addressed by the use of self-complementary backbones of AAV that have faster kinetics of expression than the single stranded version. Lastly, it would be interesting to look at the biodistribution of GALC in different parts of the brain following systemic administration of AAV in P2 or P10 mice, to determine if the time of administration impacts biodistribution in the CNS as well.

Sustained supraphysiological levels of GALC appear to be important, especially given that higher doses of AAV-GALC seem to be positively correlated with survival in animal models. Studies indicate that AAVrh10-treated Twitcher mice that show phenotypic correction have 2-fold higher levels of GALC in their brains compared to wildtype mice (124). The need for abnormally high levels of GALC is likely due to the impaired ability of GALC-deficient cells to take up extracellular GALC for cross-correction (61). However, despite the evidence supporting the idea that higher the dose, the better the overall survival, one must bear in mind that the therapeutic dose used in animal studies ultimately needs to be translated to the clinic. Therefore, while these studies are essential for establishing proof of concept, in order to avoid AAV-based toxicity, alternative methods might be necessary in order to achieve higher protein levels *in vivo* without increasing viral dose. For instance, engineering potent transgene cassettes through codon-optimization, selection of promoter, polyA termination sequences, post-regulatory transcriptional elements, and stronger signal secretion sequences are examples of features to consider. In addition, molecular engineering of the GALC enzyme can improve its secretion and uptake efficiency for subsequent cross-correction—a strategy that could be combined with gene therapy approaches. For instance, chimeric GALC with the HIV Tat protein transduction domain was shown to have a 6-fold increase in cross-correction of GALC *in vitro* (151). More recently, GALC secretion and blood brain barrier transport was enhanced by using an alternative signal secretion peptide (iduronate-2-sulfatase) and low-density lipoprotein receptor binding domain, respectively, both *in vitro* and *in vivo* (35, 152). In addition, it may be worthwhile to pursue alternative therapeutic strategies. For example, GALC rescue and knockdown of ACDase may be combined within the same vector cassette for a potentially sustained reduction in psychosine.

Finally, the nature of the synergistic improvement seen in animals receiving both gene therapy and BMT warrants further exploration. In contrast to gene therapy, the mechanism through which BMT ameliorates pathology is not as apparent. It is possible that the conditioning prior to BMT might actually be important in eliminating GALC-deficient macrophages and hence the potential pool of future inflammatory globoid cells. There may be some evidence to support this idea. In one study, BMT was combined with AAV9 administered through three different routes (IC, IT, and IV) (34). What was interesting about this study is that the authors used BMT without any immunosuppressive preconditioning, and the difference in lifespan extension in the AAV+BMT group (compared to AAV only) was a mere 3 weeks (median survival of 263 and 284 days, respectively). Moreover, the BMT-only treated mice showed macrophage infiltration in the sciatic nerve at P40, which persisted over time. In addition, another study showed that delaying BMT (from P10 to a later time such as P17) decreases median survival of the treated mice, possibly due to the irreversible inflammation or damage caused by globoid cells (126).

BMT may tone down neuroinflammation based on the status of inflammatory markers such as IL-6, TNFα, MIP-1β, and MCP-1 in the brain (33). However, it is possible that the decrease in these markers is associated with the conditioning for BMT (such as immunosuppression from preconditioning agent, busulfan), and not BMT itself. In a study in which no immunosuppressive preconditioning was used, a dramatic decrease in TNFα, CXCL1, and IL-1β was seen for AAV only compared to BMT only. However, in this study, it is also possible that the absence in these effects was associated with the low level of chimerism achieved in the BMT-treated mice (34). Further studies should help provide more insights on this observation. It is likely that BMT supplements AAV therapy by providing an additional long-term supply of GALC, though its efficiency likely varies based on the achieved chimerism. BMT-derived GALC might be particularly important at later time points, when normal cell turnover has reduced overall AAV-driven GALC expression. For example, in the same study, while no psychosine could be seen in the CNS (in the AAV9-only group at P40), the levels of psychosine were significantly increased at time of death (34). This indicates that AAV9-driven GALC in the CNS (despite being administered locally) was not sufficient to sustain lowering of psychosine levels. Moreover, Rafi et al. (126) show that using BM cells from mice heterozygous for GALC showed median survival (200 days) that was comparable to 1/2X of a dose of AAVrh10. This lends further support to the idea that BMT might be contributing to GALC cross-correction. Therefore, BMT might help support AAV therapy in three main ways. First, the conditioning preceding BMT might eliminate the source pool of inflammatory globoid cells to some degree. Second, BMT might supplement AAV therapy with a continuous supply of additional GALC in the long-term. Lastly, GALC-positive macrophages infused in the process may help support the homeostatic maintenance of myelin through phagocytosis. If the beneficial effects of BMT are actually associated more with removal of GALC-lacking myeloid progenitor cells than the infusion of GALC-positive cells, it may be worthwhile to target the hematopoietic stem cell population directly by combining two different AAV serotypes, delivered by two separate routes, such as AAV9-GALC (IC) and an AAV6-GALC (IV). Similarly, gene approaches could be considered in which myeloid progenitor cells are targeted with AAVs to express GALC at supraphysiological levels. Although such an approach would remove GALC-lacking myeloid progenitor cells (due to GALC-induced toxicity), whether the GALC expression would be sufficient for adequate cross-correction in both the CNS and PNS would need to be determined. Furthermore, these approaches could be combined with strategies to promote myeloid cell migration into the CNS.

AAV gene therapies have potentially ushered a new frontier in our ability to tackle dozens of rare diseases, including lysosomal storage disorders such as Krabbe Disease. For KD, two clinical trials using gene therapy are currently underway (both in Phase 1/2). The first of the two is PBKR03 (PassageBio), a GALC transgene in a AAVhu68 vector (AAV9 variant) delivered through the ICM route (NCT04771416). This study will have both a low and high dose in each of two age groups (1–4 and 4–9 months). The second study combines HSCT with AAV; the molecule, FBX-101 (Forge Biologics), is a GALC transgene packaged within an AAVrh10 vector and delivered IV at either a low or high dose following HSCT (NCT04693598). The results from these clinical trials, combined with results from clinical trials of other LSDs such as Metachromatic leukodystrophy (MLD), will be instrumental in helping us refine our approach to these debilitating diseases in the future. For instance, a recently concluded clinical trial using AAVrh10 in 4 children who had pre- or early-symptomatic MLD found that all treated children continued to develop symptoms associated with MLD (NCT01801709). While disappointing, we can speculate on some of the reasons that might be associated with the lack of efficacy observed in the trial. The Arylsulfatase A gene, associated with MLD, is quite similar to GALC in terms of its cellular distribution and function—both enzymes are lysosomal enzymes that process substrates important in myelin homeostasis (153). Not surprisingly, the symptoms of MLD strongly overlap with those observed in Krabbe disease, which are a combination of both CNS and PNS pathology. In the AAVrh10 clinical trial for MLD, AAVrh10-hARSA was administered to patients intracerebrally, likely resulting in negligible enzymatic cross-correction in the spinal cord or the PNS. Although ARSA activity in the CSF of treated patients reached 20–70% of control values, ARSA levels in other critical tissues may have particularly low. The results of this trial validate the hypothesis that restoring sufficient levels of the enzyme in the CNS is not sufficient in patients with peripheral neuropathy—there is a need for systemic cross-correction.

In addition to KD, clinical trials using AAV for treating several others LSDs are currently underway. These diseases include Fabry disease (FLT190 and 4D-310), late onset Pompe disease (SPK-3006, AAV2/8SPhGAA, and AT845), Mucopolysaccharidosis Type I (SB-318), Mucopolysaccharidosis Type IIIA (LYS-SAF302), Mucopolysaccharidosis Type IIIB (rAAV2/5-hNAGLU), Mucopolysaccharidosis Type VI (AAV2/8.TBG.hARSB), Gangliosidosis (Tay Sachs and/or Sandhoff disease) (AAV-GLB1, AXO-AAV-GM2, TSHA-101, and LYS-GM101), and Gaucher disease Type II (PR001). A more comprehensive overview of AAV-based clinical trials over the past several years was covered recently (154).

In summary, the advent of AAV based gene therapy has significantly expanded the realms of possibility for drug development specifically for autosomal recessive rare diseases like Krabbe disease. Additionally, the Human Genome Project and recent genome-wide association studies have made the near impossible task of annotating the human genome possible and made available the abundant knowledge that helps associate diseases with causal genetic mutations. With increasing exploration of AAVs for gene therapy, the future holds great promise in improving the lives of patients living with debilitating diseases.

## Author Contributions

GN and SA co-wrote and edited the manuscript. RC created Figure 2. GN created all the remaining figures. FE edited the manuscript. All authors contributed to the article and approved the submitted version.

## Conflict of Interest

GN, RC, FE, and SA were employed by Novartis Institutes for BioMedical Research at the time of research.

## Publisher's Note

All claims expressed in this article are solely those of the authors and do not necessarily represent those of their affiliated organizations, or those of the publisher, the editors and the reviewers. Any product that may be evaluated in this article, or claim that may be made by its manufacturer, is not guaranteed or endorsed by the publisher.
